# Conversational Therapy through Semi-Immersive Virtual Reality Environments for Language Recovery and Psychological Well-Being in Post Stroke Aphasia

**DOI:** 10.1155/2020/2846046

**Published:** 2020-08-06

**Authors:** A. Giachero, M. Calati, L. Pia, L. La Vista, M. Molo, C. Rugiero, C. Fornaro, P. Marangolo

**Affiliations:** ^1^Aphasia Experimental Laboratory-Fondazione Carlo Molo Onlus, Turin, Italy; ^2^Dipartimento di Psicologia, University of Turin, Italy; ^3^Dipartimento di Studi Umanistici, University Federico II, Naples, Italy; ^4^IRCCS Fondazione Santa Lucia, Rome, Italy

## Abstract

Aphasia is a highly disabling acquired language disorder generally caused by a left-lateralized brain damage. Even if traditional therapies have been shown to induce an adequate clinical improvement, a large percentage of patients are left with some degree of language impairments. Therefore, new approaches to common speech therapies are urgently needed in order to maximize the recovery from aphasia. The recent application of virtual reality (VR) to aphasia rehabilitation has already evidenced its usefulness in promoting a more pragmatically oriented treatment than conventional therapies (CT). In the present study, thirty-six chronic persons with aphasia (PWA) were randomly assigned to two groups. The VR group underwent conversational therapy during VR everyday life setting observation, while the control group was trained in a conventional setting without VR support. All patients were extensively tested through a neuropsychological battery which included not only measures for language skills and communication efficacy but also self-esteem and quality of life questionnairies. All patients were trained through a conversational approach by a speech therapist twice a week for six months (total 48 sessions). After the treatment, no significant differences among groups were found in the different measures. However, the amount of improvement in the different areas was distributed over far more cognitive and psychological aspects in the VR group than in the control group. Indeed, the within-group comparisons showed a significant enhancement in different language tasks (i.e., oral comprehension, repetition, and written language) only in the VR group. Significant gains, after the treatment, were also found, in the VR group, in different psychological dimensions (i.e., self-esteem and emotional and mood state). Given the importance of these aspects for aphasia recovery, we believe that our results add to previous evidence which points to the ecological validity and feasibility of VR treatment for language recovery and psychosocial well-being.

## 1. Introduction

Aphasia is one of the most socially disabling consequences post stroke [1–3] which manifests itself in about one-third of left brain-damaged people (30% of acute vs. 10-20% of chronic stroke patients [1]). The aphasic symptoms are heterogeneous varying in terms of severity and degree of involvement across the modalities of language, including the expression and comprehension of speech, reading, and writing [4]. Variation in the severity of expressive impairments, for example, may range from the patient's occasional inability to find the correct word to telegraphic and much reduced speech output [5]. Thus, persons with aphasia (PWA) experience frustration and depression since their exclusion from language-dependent activities has strong implications for many aspects of their emotional condition and social status. Indeed, language difficulties determine loss of autonomy with reduced opportunities for social exchanges with friends and for practising language skills in everyday life contexts [6]. Most aphasic patients show some degree of spontaneous recovery, most notably during the first 2–3 months following stroke onset; however, studies indicate that further improvements, even in chronic patients, are possible when they are provided with an intervention (see for review [7]). The impact and the consequential implications of having aphasia for the individuals themselves and their families highlight the importance of planning efficacious treatment methods [8, 9]. The traditional aphasia therapy approaches are largely based on compensatory strategies or repetitive training of lost functions [7]. However, although there is convincing evidence that those approaches are useful, over the last years, there has been a shift from impairment-oriented language therapy to functional approaches that train language skills in more realistic contexts. A central goal here is to facilitate the successful participation of the patients in authentic conversation by increasing communicative confidence, thus, empowering PWA to improve their quality of life [10, 11]. Accordingly, the latest Cochrane review on speech and language therapy following stroke concluded that therapy should enhance functional communication in ecological contexts [7]. Indeed, a common observation regarding PWA is that they can communicate much more than their linguistic abilities would suggest. Therefore, the hypothesis has been advanced that a more ecological approach aimed at restoring the patient's ability to communicate in different daily contexts would be proved useful in rehabilitation [12–15]. Within this approach, conversational therapy is one such treatment [12–16]. The main objective of this approach is to set up a natural conversation between the therapist and the PWA, a condition of communicative exchange, in which both speakers participate using their available communicative resources [14, 15]. Within this therapeutic approach, not only language but also any intentional action (e.g., gesturing, drawing) can be used to communicate. The therapeutic goal shifts from a purely analytic treatment aimed at the recovery of the damaged linguistic processes, still used in the traditional approach, to a global approach. The latter considers the ability of the PWA to communicate as a whole through strengthening his/her residual communicative functions [12–16].

In these last years, scientific advancements in language conceptualization and the progress of new technologies have made new tools available for professional therapists and educators. Digital technologies offer exciting opportunities to PWAs who live with long-term communication deficits (see for review [17]). Among these technologies, computer therapies deliver individually tailored exercises for training a range of language skills, including word retrieval [18, 19], sentence building [20, 21], and language comprehension [22]. The StepByStep (PLOS) computer program includes over 10,000 language exercises ranging from listening to writing words or producing sentences [17, 19]. It was shown that patients who received StepByStep training achieved greater improvement in naming ability compared with patients who received the standard speech and language therapy [19]. A study that investigated Multicue as a rehabilitation program demonstrated significant improvement in naming abilities measured through the Boston Naming Test in patients who received the training; however, no significant improvement was shown in verbal communication skills [18, 23, 24]. Overall, these studies suggested that independent computerized therapies can be as effective as clinician-guided therapies [24]. However, most of these studies exhibited a positive effect on word finding in picture naming tasks but not on communicative abilities [18, 23, 24]. Additionally, iPad-based aphasia rehabilitation treatments have been investigated but, as for computer therapies, most of the findings investigated the impact only on language functions [25–28].

Among the applied technologies, an area that particularly merits exploration is virtual reality (VR). Development of VR applications for rehabilitation of aphasia is still in its early stages ([29–32]; see for a review [17]). This involves a computer-generated simulation of 3D environments with which the user can experience a semi-immersive interaction that may encourage language practice in real context communication settings. Typically, an individual entering a virtual environment feels a part of this world and he/she has the opportunity to interact with it almost as he/she would do in the real world. Uses of VR in healthcare are widespread, ranging from the treatment of physical impairments [33, 34], post traumatic stress disorders [35], and anxiety [36, 37]. Virtual reality applications have been also explored on different communication disorders such a speaking phobias [38], stuttering [39], and autism [40, 41]. However, to date, the use of VR for language recovery in aphasia has been limited. Stark et al. [42] developed a virtual house to promote individual language practice. In Aphasia Script [43], therapy is based on the oral production of scripts, which are short functional dialogs structured around communication of everyday activities. Script treatment can be delivered by a virtual therapist (VT) through a computer or by a real therapist. A randomized controlled cross-over study using Aphasia Script was conducted to investigate the effect of high or low cuing on treatment outcomes over time [43]. Eight participants were recruited and randomized to receive intensive computer-based script training differing in the amount of high or low cuing provided during treatment. In the high cue treatment condition, participants could hear the virtual therapist (VT) during listening, choral reading, and reading aloud, with auditory cues (therapist speaking) and visual cues (therapist's mouth movements) available at the start, during, and after practice. In the low cue condition, they received visual and auditory cues when listening to the script being read aloud initially and after practice, but did not receive auditory and visual support during sentence practice.

Performance was measured by averaging the sentence level word accuracy of participants' production of ten sentences (ten words in length) during each assessment session. Accuracy of words were rated using a previously validated six-point scale, and the overall session score expressed on a scale from 0 to 100%. Training resulted in significant gains in script acquisition with maintenance of skills at three and six weeks posttreatment. Differences between cuing conditions were not significant. Three weeks of computer-based script training resulted in increased accuracy and rate of script production. The mean baseline performance was 50.0 (26.4)% for accuracy and 23.7 (20.6) for rate (words per minute, WPM). At the end of training, it had improved to 77.8 (19.6)% and 60.3 (30.5) WPM for accuracy and rate, respectively. Moreover, although there was a slight drop in performance noted at both three weeks and six weeks posttreatment, the decreases were small. At three weeks posttreatment, the mean scores for accuracy were 72.2 (22.4) and the mean scores for rate were 55.2 (34.0). By six weeks posttreatment, these scores had declined slightly to 68.6 (24.7) for accuracy and 51.4 (35.8) for rate [43].

The Web Oral Reading for Language in Aphasia (ORLA, Rehabilitation Institute of Chicago) [44] is a therapy program where patients repeatedly read aloud sentences, first in unison with a clinician and then independently. The program was developed to improve the patient's reading comprehension skills by providing practice in phonological and semantic reading routes. Following a no-treatment period, twenty-five individuals with chronic nonfluent aphasia were randomly assigned to receive twenty-four sessions of ORLA, 1–3 times per week, either by computer (*N* = 11) or by a speech language pathologist (*N* = 14) (SLP-ORLA). Results showed that the mean change in the Western Aphasia Battery-Aphasia Quotient scores (the primary outcome measure) from pre- to posttreatment was 3.29 (SD = 6.16) for the eleven participants receiving computer ORLA. In comparison, the mean change during the no-treatment phase from baseline to posttreatment was only −0.4. Student *t*-tests were used to compute the change from pretreatment to posttreatment between the computer ORLA and SLP-ORLA groups. No significant differences were found on any of the outcome measures (*P* values ranged from 0.2 to 0.6), suggesting good compatibility and feasibility of the VR version [45].

Sentactics (Sentactics Corporation, Concord, CA, USA) is a linguistic treatment which aims at improving sentence production and comprehension deficits through a virtual clinician. Patients are trained repeating and reading sentences and describing pictures presented on the screen. Thompson et al. [20] conducted a study to test the efficacy of Sentactics as an aphasia rehabilitation tool. Computer-delivered Sentactics was compared with a clinician-delivered therapy. Results showed that patients who received Sentactics training significantly improved in production and comprehension for both trained (0% to 90% production, 0% to 30% comprehension) and untrained sentences (0% to 30% production, 0% to 15% comprehension) [20].

More recently, a multiuser virtual world called EVA Park was designed for PWA. The authors wanted to investigate whether virtual environments would enable people with moderate aphasia to practice speech successfully with one or more conversational partners [32]. The results collected in twenty PWA, after five weeks of therapy intervention, revealed that the VR experience offered participants rich insights into aspects which go beyond the therapeutic outcomes. Indeed, PWAs experienced conversational initiative, positive emotional, and social outcomes and their therapeutic benefits were well-maintained on a measure of everyday communication (mean scores across the three time points: week 1: 6.5 vs. week 7: 7.2 vs. week 13: 7.4, Communication Activities of Daily Living (CADL-2) test). However, as also observed by the authors [32], one limitation of their study was related to the lack of a control group inclusion which should have undergone a different treatment. This allows no conclusions to be drawn about the relative merits of the therapy delivered in VR compared to “conventional” face to face therapy.

Kurland et al. [46] investigated the effects of a tablet-based home practice program with telepractice on treatment outcomes in twenty-one individuals with chronic aphasia. The main outcome measure was percent accuracy on naming sets of treated and untreated objects and actions. Overall, results showed that home practice was effective for all participants with severity moderating treatment effects, such that individuals with the most severe aphasia made and maintained fewer gains (difference between post- and pretreatment in naming accuracy, severe: 0.067 vs. moderate: 0.057 vs. mild: 0.123 for treated items; severe: 0.099 vs. moderate: 0.157 vs. mild: 0.138 for untreated items).

Marshall et al. [47] reported two single case studies exploring the impact of daily language stimulation delivered through EVA Park platform [32] for treated and untreated word production, connected speech, and functional communication. After the therapy, outcomes varied across the different test measurements. The noun therapy significantly improved the naming of treated words in case study 1 but not in case study 2 (case 1, pre-posttreatment: 25 out of 50 items vs. 44 out of 50 items), with good maintenance after five weeks (case 1, 41 out of 50 items). There was no generalisation to untreated words (case 1, pre-posttreatment 27 out of 50 items vs. 25 out of 50 items), connected speech, or functional communication.

Within a case series (*N* = 3), Carragher et al. [48] explored the effect of storytelling intervention delivered in EVA Park [32]. The intervention dose was four sessions per week for a total of five weeks (twenty hours total). Following intervention, two participants (“Ange” and “Sally”) showed substantial increases in the percentage of correct content words produced (Ange: 36.5%; Sally: 35.5%). The third participant demonstrated a more modest change with an increase of 12.1%.

Very recently, Palmer et al. [49] reported the first multicentre randomised controlled trial (BIG CACTUS) in patients with post stroke chronic aphasia (>6 months) to assess both the clinical and cost-effectiveness of self-managed computerised speech and language therapy (CSLT). Two hundred and seventy-five participants were randomly assigned to either six months of usual care (usual care group, *N* = 101), daily self-managed CSLT plus usual care (CSLT group, *N* = 97), or attention control plus usual care (attention control group, *N* = 80). Coprimary outcomes were changes, between baseline and 6 months after randomization, in lexical retrieval of personally relevant words in a picture naming test and in functional communication ability measured with the use of Therapy Outcome Measures (TOMs). The key secondary outcome was change in self-perception of communication and social participation measured through the Communication Outcomes After Stroke (COAST) questionnaire self-rated by the patient. Word finding improvement was 16. 2% higher in the CSLT group than in the usual care group and 14. 4% higher than in the attention control group. Improvement in word finding was maintained 6 months after the intervention period. However, CSLT did not have an effect on conversation, self-perceived improvements in everyday communication, social participation, and quality of life [49].

Maresca et al. [50] employed a VR tablet in order to evaluate the effectiveness of a rehabilitation training for aphasia. Thirty PWA were randomly assigned into either the control or the experimental group. The study lasted six months and included two phases. During the first phase, the experimental group was trained through the VR tablet, while the control group underwent traditional therapy. In the second phase, the experimental group was discharged but it was provided with the VR tablet, while the control group was assigned to community services. Results showed that the experimental group improved in all investigated tasks except in writing, while the control group improved only in comprehension, depression, and quality of life.

In summary, although in the field of aphasia rehabilitation, technical devices have begun to be employed, to date, digital versions of traditional language therapy exercises have been mostly used [17]. Very few studies have explored digital applications, including VR settings, for conversation in social interaction (but see [33, 51, 52]). More importantly, none of the cited studies has investigated the impact of VR technology on the patient's psychological well-being [but see 49].

Here, we report a video-based conversational training approach which makes use of semi-immersive VR environments to investigate their therapeutic benefits in enhancing language skills, communication efficacy, and psychosocial aspects (i.e., the self-esteem level; the patient's emotional, health, and humoral states) in a group of eighteen nonfluent chronic PWA. The efficacy of the VR approach was compared to the results of a matched control group of eighteen PWA who underwent the same conversational training without VR support.

### 1.1. Aims

The study addressed the following research questions (RQs):

*RQ1:* does conversational therapy delivered via semi-immersive VR environments enhance language recovery in chronic post stroke aphasia?

*RQ2:* do therapy benefits generalize to measures of communication efficacy and psychological well-being?

*RQ3:* is VR therapy equivalent or more effective than conventional training?

### 1.2. Hypothesis

In line with previous literature [8, 13, 14] which suggests that language treatment should enhance functional communication in ecological contexts, we hypothesize that conversational therapy combined with VR would be effective for aphasia. Since a central aspect of conversational approach is to set up communicative exchanges between the therapist and the patient in ecological contexts [15, 16], we further believe that treatment benefit would generalize to communication efficacy and, possibly, to psychological well-being.

## 2. Materials and Methods

### 2.1. Participants

All patients were recruited from the neurological departments of different hospitals in Turin. Seventy-six have completed their speech therapy cycle and contacted the Experimental Laboratory of Aphasia of the Fondazione Carlo Molo Onlus in Turin in order to participate as volunteers in the research. A preliminary neuropsychological assessment was handled by an independent neuropsychologist who was blinded to the research. The inclusion criteria were fluent users of Italian, premorbidly right handed, a diagnosis of aphasia due to a single left hemisphere stroke occurring more than six months prior to the study; absence of cognitive impairment; ability to follow instructions; no hemispatial neglect; no articulatory disorder; no uncorrected visual impairment (self-report); and no hearing loss (screened via pure tone audiometry). Since our treatment was based on a conversational therapy approach aimed at enhancing verbal communication, we selected only nonfluent patients. Patients were not enrolled if they had a premorbid speech and language disorder caused by a neurological deficit other than stroke. Twenty patients were excluded because they did not meet the criteria. Fifteen people gave up for logistic reasons. Five had another stroke during the enrollment period. The thirty-six patients selected were randomly assigned to two different conditions by a researcher not involved in the research, using the Research Randomizer (https://www.randomizer.org/), a free web-based service that offers instant random sampling and random assignment. All have age between 32 and 77 years (59.75+/-11.21) with an educational level of 5 to 18 years (11.25+/-3.54). Eighteen patients were assigned to the experimental group and eighteen to the control group. In order to obtain more accurate results, the study included a sample size that would allow parametric statistics to be applied to the data.

Table 1 provides background details for the participants.

### 2.2. Ethical Approval

The data analysed in the current study conformed with the Helsinki Declaration. Our named Institutional Review Board (Ethical Committee, University of Turin) specifically approved this study (protocol 100960) with the understanding and written consent of each subject.

### 2.3. Materials and Apparatus

The semi-immersive VR scenarios were projected through a screen (50 inches). They were created with a NeuroVR 2.0 open source software (http://www.neurovr2.org) by the authors of the present study from the Afasia Experimental Laboratory of Carlo Molo Onlus Foundation in Turin. In order to favour the interaction between patients within the therapeutic setting, the authors opted for a semi-immersive virtual reality condition in which no patient wore a helmet. To limit the window effect typical of a nonimmersive virtual reality condition, a 50-inch curved screen was used to guarantee a sufficient level of image depth and sense of immersion for each patient [53]. The apparatus projects different virtual scenarios that can be explored by the patient. In order to elicit the ecological validity of the VR settings, each scenario represented everyday communication situations, such as different environments inside a city (i.e., a supermarket, a restaurant, an amusement park, the station, and the post office) (see Table 2). The apparatus was set up in order to integrate each scenario with different cognitive exercises, such as language (i.e., phonology, lexicon, semantics, and grammar), memory (i.e., working memory), attentional (i.e., sustained attention, selective attention), and executive function tasks. Cognitive exercises range from the simplest to the most complex ones. For example, the language tasks could require the patient to select the correct word among phonological (i.e., cappello (hat) and carrello (trolley)) or semantic (valigia (suitcase) and borsa (bag)) distractors, while the executive functions tasks involve the patient to manage unexpected events (see Table 2).

The interaction among patients was mediated by a speech therapist who operated in the VR scenario through the use of a personal computer. As the patient selects a virtual scenario (e.g., supermarket), the therapist presses the keyboard allowing the patient to explore it. Thus, the Neuro VR does not provide for the patient to explore the virtual environments without the help of the therapist. Within each scenario, different choices can be made by the patient (i.e., in the “Travel” scenario, patients could decide which sport to play (tennis or golf)). As the patient communicates to the therapist his/her choice, the therapist moves the mouse by clicking on the option chosen by the patient, thus opening a new screen in which the selected chooses appears. For example, if the patient chooses a tennis court, he/she can move the tennis ball by naming the objects placed on the side of the ball. Then, if the objects are correctly named, the therapist throws the ball to the other side. The response shift will thus be transferred to the patient who is playing together at that time and who, in turn, must name the objects placed on the other side of the field. Each virtual scenario includes the same number of cognitive exercises which train the different functions and the exercises vary in number and difficulty on the basis of the itinerary chosen. Thus, the apparatus automatically selects the exercises to be performed as the patient gets through the virtual environment and makes his/her choice (i.e., if the patient is at the greengrocer, he may be asked to indicate a fruit among semantic or phonological distractors or to perform a category fluency task).

**Figure 1:**
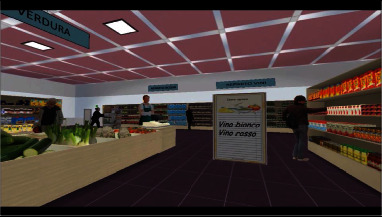
ScenePlayer NeuroVR: “Supermarket”.

For example, in the Supermarket scenario (see Figure 1), the first objective is to get groceries. Thus, the patient is asked to select the food from the shelves and to perform a concomitant cognitive exercise, such as a semantic fluency task (i.e., “tell me all you need to get groceries” ➔ money, wallet, bag, credit card, and so on). The different tasks are graded by difficulty. Thus, the item selection may result more or less difficult by the presence of phonological (i.e., cappello (hat) and carrello (trolley)) or semantic (valigia (suitcase) and borsa (bag)) distractors. If the patient is not able to retrieve the word, he/she can be facilitated through visual or verbal cues. The different cognitive exercises alternate with conversational phases in which the patient is asked to describe the scenario, to request information to the people which appear in the scenario (i.e., a policeman), to conversate with the speech therapist or the other patients available in the therapy room. The scenario can also sometimes present some contingencies that the patient has to face (i.e., a robbery to a lady ➔ calling the policeman).

### 2.4. Procedure

The 36 participants were randomly assigned through a computer software program to one of two training: (1) conversational training combined with VR (*N* = 18) and (2) conversational training without VR (conventional therapy (CT)) (*N* = 18). Assessments were administered by an independent neuropsychologist who was blinded to the condition under which the patient was assigned. Within each condition, in order to facilitate their interaction, patients were organised into six groups of three people. Due to resource constraints, it was not feasible to run more than 3 participants simultaneously. For each training, participants completed twenty-four weeks of intensive language training (total=six months), each treatment lasted two hours, and it was performed twice a week.

### 2.5. Outcome Measures

A range of outcome measures was used to evaluate the effects of the two treatments (VR vs. CT). Language, communication skills, and psychosocial aspects were tested before and after the training via standardized test batteries. The primary outcome language measure was the Aachen Aphasia Test (A.A.T.) [54].

Secondary outcome measures included the Conversation Analysis Profile for People with Aphasia test (C.A.P.P.A. test, [55]) which evaluates the patient's communication skills both from the patient and of his/her caregiver perspective. The questionnaire investigates the frequency and the severity of the patient's communicative disorder with respect to four different areas: language ability, self-correction, verbal initiative, and turn taking and topic management. Two different batteries for evaluating the psychosocial aspects of the patient's disability such as the Visual Analogue Self Esteem Scale [56] and the W.H.O.Q.O.L. Scale [57] were also included. Subjects were also administered memory tests (i.e., digit span, cut − off scores < 5), the Corsi test (cut − off scores < 4) and attentional tasks (attentional matrices, cut-off scores ≤ 30) [58], and the Trail Making test (*B*-*A* seconds, cut-off scores > 186) [59]), which excluded the presence of working memory and attention deficits that might have confounded the data. All subjects were classified nonfluent aphasics because of their reduced spontaneous speech with short sentences and frequent word-finding difficulties. They had no articulatory deficits that might have distorted their oral production.

### 2.6. Conversational Training with VR

The six-month training consisted of two-hour therapy sessions twice a week for twenty-four weeks (total = 48 hours of therapy). At the beginning of each session, the three patients jointly chose a virtual scenario (i.e., the restaurant) and the therapist moved the mouse by clicking on the chosen option and, thus, starting the sequence of events shown in the scenario. The participants were required to observe each VR environment and to come up with a dialogue with the help of the therapist. If no patient took the initiative to speak, the therapist described the situation and then asked each patient for some information about the scenario (e.g., which hotel location to select on a map displayed in the scenario). Then, each participant had to provide a feedback (e.g., repeat what he/she has understood) to the patient who has previously spoken. During the treatment, all patients had to perform both conversational therapy and cognitive exercises. For example, after completing the “Railway Station” block, which requires limited interaction among patients, the patients come to the “Travel” block which facilitates conversational exchanges and positive competition among participants (i.e., in the “Travel block,” patients have to decide where they want to lodge (i.e., camping, hotel, camper) and each patient should convince the others about the best accommodation by trying to make his/her preference prevails; patients can also make a price estimation with respect to the different ways of travelling. Those who come closest to the correct price get the prevalence). In accordance with the principle of the Conversational Therapy approach [15, 16], no formal protocol is directed the therapist. The therapy adapted from time to time to the patient's needs, and the exercises went on based on the patient's responses. It was not possible to continue to the next block if all tasks of the previous one were not carried out. The main objective of the therapy was to set up a natural conversation on the virtual scenario in which all interlocutors participated using their available communicative resources. Both the patients and the therapist were left free to use verbal or nonverbal communication (e.g., orthographic or phonological cues, gestures, and drawings). This possibility of using any communication means was also supported by a whiteboard on which the patient could draw or write. The whiteboard was also used as a support for cognitive exercises (e.g., in a naming task, the therapist could write on the whiteboard the first syllable of a word (orthographic cue) in order to facilitate lexical retrieval). The therapist had to accept all the information provided by the patient and try to relate it to the topic of conversation in order to improve its content and informativeness. The goal of the therapy was to enhance verbal communication, to make the patient more informative day-by-day with the context, and to enable him/her to talk about the video without the therapist's support. In order to facilitate communicative exchanges among patients, the three participants were half-moon seated. Thus, they were each watching the screen and at the same time interacting between each other in the room. Each patient, in turn, was required to take the floor.

### 2.7. Conversational Training without VR

The procedure and the training were the same as the one for the experimental group but without the VR scenarios. A total of six months of training and two-hour therapy sessions twice a week (total = 48 hours of therapy) were provided to each patient. During this treatment, patients were involved in cooperative conversations regarding different topics (i.e., hobbies, job, and holidays; what have you done during the week-end?) [14, 15]. As for the VR training, the conversation alternated with cognitive exercises but only with the support of the whiteboard.

### 2.8. Data Analysis

All statistical analyses were conducted with IBM SPSS Statistics 22 software. For the outcome measures, two ANOVA analyses were planned. The first was a mixed ANOVA with the within variable of time (two levels: pre vs. posttreatment), and the between variable of group (two levels: VR group vs. control group). This directly compared the results of the two groups at two time points (pre vs. posttreatment) on each test. The second analysis was a within-group ANOVA, with the within-variable TIME (two levels: pre vs. posttreatment) comparing, within each group separately, the mean scores at two time points on each test. If the ANOVA showed significant effects, respective post hoc Bonferroni tests were conducted. In order to investigate baseline differences between the two groups, one-way ANOVA comparisons for age, educational level, time post stroke, and screening measures were also applied. Since within each group, subjects were treated in groups of three, we had six comparisons, thus, the significance level was set at *α* 0.008 in all statistical analyses. To evaluate the extent of the effects for each variable, the values of the effect size were entered using partial *η*^2^ index, which SPSS software automatically associates with ANOVA.

## 3. Results

One-way ANOVA comparisons for age, educational level, time post stroke, and screening measures found significant baseline differences between the groups only with respect to time post stroke (*F* (1.34) = 14.186, *P* = 0.001). Indeed, the time post stroke of the VR group (mean = 36.33, DS: 9.86) was significantly shorter than that of the control group (mean = 49.17, DS: 10.57).

### 3.1. Outcome Measures

#### 3.1.1. Aachen Aphasia Test

The mixed ANOVA revealed the main effect of time: token test (*F*(1, 34) = 12.386, *p* = 0.001, partial *η*^2^ = 0.267); repetition (*F*(1, 34) = 27.092, *p* < 0.001, partial *η*^2^ = 0.443); written language (*F*(1, 34) = 18.417, *p* < 0.001, partial *η*^2^ = 0.351); naming (*F*(1, 34) = 9.177, *p* = 0.005, partial *η*^2^ = 0.213); and comprehension (*F*(1, 34) = 11.098, *p* = 0.002, partial *η*^2^ = 0.246).

No effect of GROUP and no interaction time^∗^group for each AAT subtest were found.

So, participants improved between pre- and posttreatment on this measure, but both groups improved equally.

**Figure 2:**
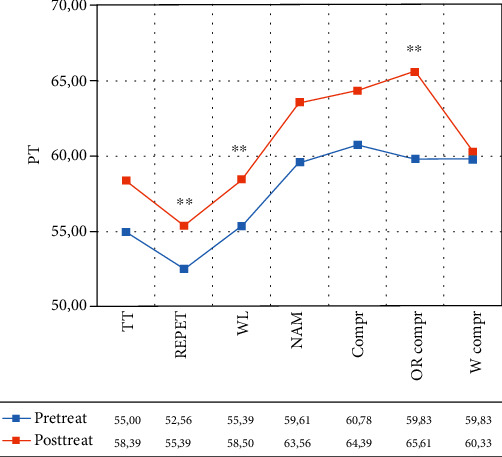
Normalized scores in the different subtests of the Aachen Aphasia Test (AAT) for the VR group. Legend: Pre-Treat: pretreatment; Post-Treat: posttreatment; TT: token test, REPET: repetition; WL = written Language; NAM: naming; Compr: comprehension; OR Compr: oral comprehension; W Compr: written comprehension; PT: normalized scores; Within-group ANOVA: ^∗^*p* ≤ 0.008.

In the VR group, the within-group ANOVA showed a significant effect of time in repetition (*F*(1, 12) = 15.211, *p* = 0.002, partial *η*^2^ = 0.559, mean = 52.56 (DS:8.09) pretreatment vs. 55.39 (DS: 10.07) posttreatment); written language (*F*(1.12) = 14.792, *p* = 0.002, partial *η*^2^ = 0.552, mean = 55.39 (DS:10.98) pretreatment vs. 58.50 (DS:10.82) posttreatment; oral comprehension (*F*(1, 12) = 10.291, *p* = 0.008, partial *η*^2^ = 0.462, mean = 59.83 (DS:9.33) pretreatment vs. 65.61 (DS: 10.72) posttreatment) (see Figure 2 and Table 3). According to the AAT cut-off score, before the treatment, for each subtest, patients were classified as moderate aphasics. After the treatment, they were still below the cut-off score but they were classified as mild aphasics (see Figure 2).

**Figure 3:**
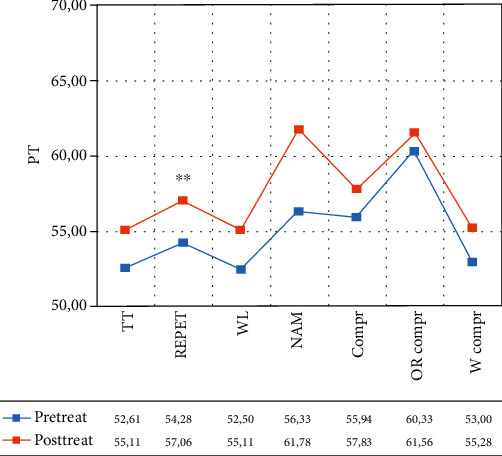
Normalized scores in the different subtests of the Aachen Aphasia Test (AAT) for the control group. Legend: Pre-Treat: pretreatment; Post-Treat: posttreatment; TT: token test, REPET: repetition; WL = written language; NAM: naming; Compr: comprehension; OR Compr: oral comprehension; W Compr: written comprehension; PT: normalized scores; within-group ANOVA: ^∗∗^*p* ≤ 0.008.

In the control group, the within-group ANOVA showed a significant effect of time only in repetition (*F*(1, 12) = 12.255, *p* = 0.004, partial *η*^2^ = 0.505; mean = 54.28 (DS: 9.55) pretreatment vs. 57.06 (DS: 9.73) posttreatment (see Figure 3 and Table 3). According to the AAT cut-off score, before the treatment, for each subtest, patients were classified as moderate aphasics. After the treatment, they were still below the cut-off score but they were classified as mild aphasics (see Figure 3).

#### 3.1.2. C.A.P.P.A. Test—Patient's Perspective

The mixed ANOVA revealed the main effect of time from the patient's perspective: language ability for frequency (*F*(1, 34) = 21.564, *p* < 0.001, partial *η*^2^ = 0.388) and severity (*F*(1, 34) = 25.326, *p* < 0.001, partial *η*^2^ = 0.427), self-correction for severity (*F*(1, 34) = 9.491, *p* = 0.004, partial *η*^2^ = 0.218), turn taking for frequency (*F*(1, 34) = 13.209, *p* = 0.001, partial *η*^2^ = 0.280) and severity (*F*(1, 34) = 18.570, *p* < 0.001, partial *η*^2^ = 0.353), and topic management for frequency (*F*(1, 34) = 17.585, *p* < 0.001, partial *η*^2^ = 0.341) and severity (*F*(1, 34) = 13.401, *p* = 0.001, partial *η*^2^ = 0.283).

No effect of group and no interaction time∗group for each C.A.P.P.A. subtest were found.

So participants improved between pre- and posttreatment on this measure from the patient's perspective, but both groups improved equally.

**Figure 4:**
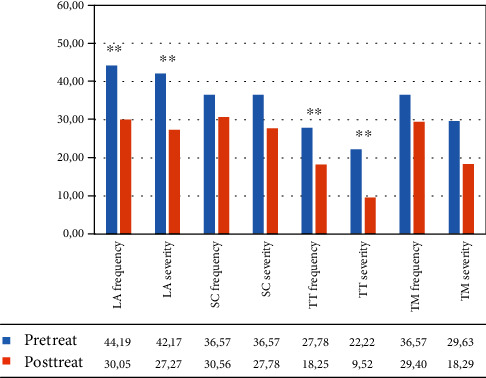
Mean percentage of scores in the different subtests of the C.A.P.P.A. test, for frequency and severity, in the VR Group from the patient‘s perspective. Legend: Pre-Treat: pretreatment. Post-Treat: posttreatment; LA: language ability; SC: ability to self-correct; TT: turn taking; TM: topic management; within-group ANOVA: ^∗∗^*p* ≤ 0.008.

In the VR group, the within-group ANOVA showed a significant effect of time: language ability (*F*(1, 12) = 19.969, *p* = 0.001, partial *η*^2^ = 0.625; mean = 44.19 (DS: 21.54) pretreatment vs. 30.05 (DS: 16.95) posttreatment for frequency; *F*(1, 12) = 27.844, *p* < 0.001, partial *η*^2^ = 0.699; mean = 42.17 (DS: 24.84) pretreatment vs. 27.27 (DS: 22.21) posttreatment for severity) and turn taking (*F*(1, 12) = 13.394, *p* = 0.003, partial *η*^2^ = 0.527; mean = 27.78 (DS: 18.46) pretreatment vs. 18.25 (DS: 16.66) posttreatment for frequency; *F*(1, 12) = 51.209, *p* < 0.001, partial *η*^2^ = 0.810; mean = 22.22 (DS: 20.04) pretreatment vs. 9.52 (DS: 12.49) posttreatment for severity) (see Figure 4 and Table 4).

**Figure 5:**
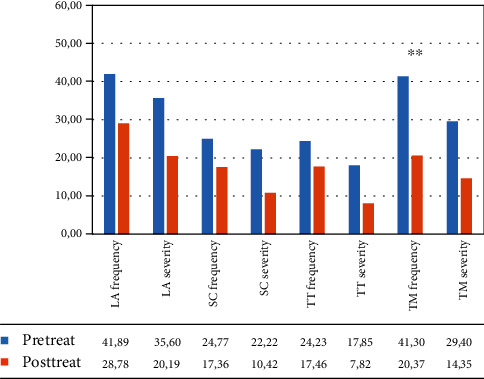
Mean percentage of scores in the different subtests of the C.A.P.P.A. test, for frequency and severity, in the control group from the patient's perspective. Legend: Pre-Treat: pretreatment; Post-Treat: posttreatment; LA: language ability; SC: ability to self-correct; TT: turn taking; TM: topic management; within-group ANOVA: ^∗∗^*p* ≤ 0.008.

In the control group, the within-group ANOVA revealed a significant effect of time only in the frequency of the topic management subtest (*F*(1, 12) = 26.065, *p* < 0.001, partial *η*^2^ = 0.685, mean = 41.30 (DS: 26.13) pretreatment vs. 20.37 (DS: 17.83) posttreatment) (see Figure 5 and Table 4).

#### 3.1.3. C.A.P.P.A. Test—Caregiver's Perspective

The mixed ANOVA revealed the main effect of time from the caregiver's perspective: language ability, frequency (*F*(1, 34) = 30.033, *p* < 0.001, partial *η*^2^ = 0.476) and severity (*F*(1, 34) = 42.986, *p* < 0.001, partial *η*^2^ = 0.566); self-correction, frequency (*F*(1, 34) = 31.314, *p* < 0.001, partial *η*^2^ = 0.487); and severity (*F*(1, 34) = 18.257, *p* < 0.001, partial *η*^2^ = 0.356); and turn taking, frequency (*F*(1, 34) = 14.082, *p* = 0.001, partial *η*^2^ = 0.299).

No effect of group and no interaction time∗group for each C.A.P.P.A. subtest were found.

So participants improved between pre- and posttreatment on this measure from the caregiver's perspective, but both groups improved equally.

**Figure 6:**
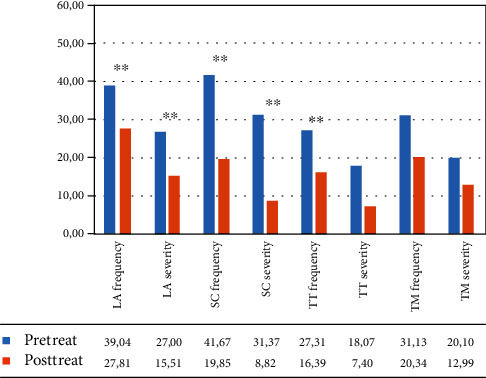
Mean percentage of scores in the different subtests of the C.A.P.P.A. test, for frequency and severity, in the VR group from the caregiver's perspective. Legend: Pre-Treat: pretreatment; Post-Treat: posttreatment; LA: language ability; SC: ability to self-correct; TT: turn taking; TM: topic management; within-group ANOVA: ^∗∗^*p* ≤ 0.008.

In the VR group, the within-group ANOVA showed a significant effect of time: language ability (*F*(1, 12) = 31.277, *p* < 0.001, partial *η*^2^ = 0.740; mean = 39.04 (DS: 15.83) pretreatment vs. 27.81 (DS: 16.85) posttreatment frequency; *F*(1, 12) = 23.076, *p* = 0.001, partial *η*^2^ = 0.677; mean = 27.00 (DS: 23.37) pretreatment vs. 15.51 (DS: 17.61) posttreatment severity); self-correction (*F*(1, 12) = 19.031, *p* = 0.001, partial *η*^2^ = 0.634; mean = 41.67 (DS: 24.52) pretreatment vs. 19.85 (DS: 17.15) posttreatment frequency; *F*(1, 12) = 15.073, *p* = 0.003, partial *η*^2^ = 0.578; mean = 31.37 (DS: 33.56) pretreatment vs. 8.82 (DS: 14.50) posttreatment severity), and turn taking (*F*(1, 12) = 21.576, *p* = 0.001, partial *η*^2^ = 0.662; mean = 27.31 (DS: 16.99) vs. 16.39 (DS: 13.07) posttreatment frequency) (see Figure 6 and Table 5).

**Figure 7:**
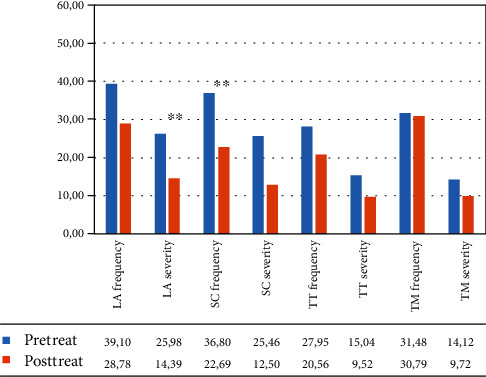
Mean percentage of scores in the different subtests of the C.A.P.P.A. test, for frequency and severity, in the control group from the caregiver‘s perspective. Legend: Pre-Treat: pretreatment; Post-Treat: posttreatment; LA: language ability; SC: ability to self-correct; TT: turn taking; TM: topic management; within-group ANOVA: ^∗∗^*p* ≤ 0.008.

In the control group, the within-group ANOVA showed a significant effect of time in language ability for severity (*F*(1, 12) = 19.062, *p* = 0.001, partial *η*^2^ = 0.614; mean = 25.98 (DS: 16.60) pretreatment vs. 14.39 (DS: 12.59) posttreatment and in self-correction for frequency (*F*(1, 12) = 18.432, *p* = 0.001, partial *η*^2^ = 0.606; mean = 36.80 (DS: 19.13) pretreatment vs. 22.69 (DS: 14.16) posttreatment (see Figure 7 and Table 5).

#### 3.1.4. Visual Analogue Self-Esteem Scale (VASES)

The mixed ANOVA revealed the main effect of time (*F*(1, 34) = 14.848, *p* < 0.001, partial *η*^2^ = 0.304) but no effect of group and no interaction time∗group.

So participants improved between pre- and posttreatment on this measure, but both groups improved equally.

Only in the virtual group, the within-group comparison showed a significant effect of time (*F*(1, 12) = 12.598, *p* = 0.004, partial *η*^2^ = 0.512; mean = 37.00 (DS: 5.46) pretreatment vs. 42.50 (DS: 6.31) posttreatment.

#### 3.1.5. WHOQoL Questionnaire

The mixed ANOVA revealed the main effect of time: WHO physical area (*F*(1, 34) = 12.622, *p* = 0.001, partial *η*^2^ = 0.271), WHO social area (*F*(1, 34) = 12.027, *p* = 0.001, partial *η*^2^ = 0.261), and WHO environmental area (*F*(1, 34) = 18.309, *p* < 0.001, partial *η*^2^ = 0.350).

No effect of group and no interaction time∗group were found.

So participants improved between pre- and posttreatment on the different scales of the WHOQoL questionnaire, but both groups improved equally.

**Figure 8:**
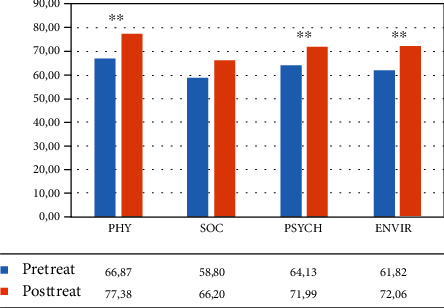
Mean percentage of scores in the different subtests of the WHOQoL questionnaire (Word Health Organization Quality of Life—WHOQOL group, 1998) for the VR group. Legend: Pre-Treat: pretreatment; Post-Treat: posttreatment; PHY: physical; SOC: social; PSYCH: psychological; ENVIR: environmental; within-group ANOVA: ^∗∗^*p* ≤ 0.008.

In the VR group, the within-group ANOVA revealed a significant effect of time in different areas: WHO physical area (*F*(1, 12) = 15.030, *p* = 0.002, partial *η*^2^ = 0.556; mean = 66.87 (DS: 11.86) pretreatment vs. 77.38 (DS: 14.55) posttreatment), WHO psychological area (*F*(1, 12) = 18.578, *p* = 0.001, partial *η*^2^ = 0.608; mean = 64.13 (DS: 15.34) pretreatment vs. 71.99 (DS: 14.85) posttreatment), and WHO environmental area (*F*(1, 12) = 30.865, *p* < 0.001, partial *η*^2^ = 0.720; mean = 61.82 (DS: 13.50) pretreatment vs. 72.06 (DS: 13.32) posttreatment) (see Figure 8 and Table 6).

**Figure 9:**
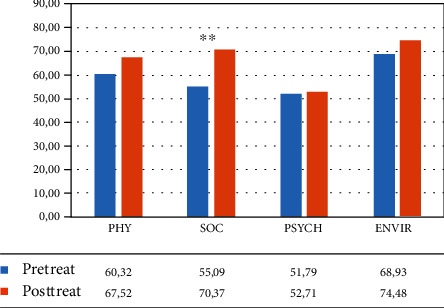
Mean percentage of scores in the different subtests of the WHOQoL questionnaire (Word Health Organization Quality of Life–WHOQOL group, 1998) for the control group. Legend: Pre-Treat: pretreatment; Post-Treat: posttreatment; PHY: physical; SOC: social; PSYCH: psychological; ENVIR: environmental; within-group ANOVA: ^∗∗^*p* ≤ 0.008.

In the control group, the within-group ANOVA revealed a significant effect of time only in the social area (*F*(1, 12) = 13.271, *p* = 0.003, partial *η*^2^ = 0.525; mean = 55.09 (DS: 17.42) pretreatment vs. 70.37 (DS: 14.92) posttreatment) (see Figure 9 and Table 6).

## 4. Discussion

The present study investigated the usefulness of semi-immersive virtual environments combined with a conversational therapy approach for enhancing language recovery in a sample of post stroke chronic PWA. It employed a randomized controlled design which compared the results of eighteen PWA who received an intensive VR intervention combined with conversational therapy with the performance of eighteen matched controls who underwent the same conversational therapy but without VR. A broad range of outcome measures examined the impact of the two treatments (VR vs. without VR) not only on language-specific tasks (AAT test) but also on the patients' communication abilities (C.A.P.P.A. test) and on different psychosocial aspects measured through the VASES and WHOQoL. The study showed that substantial improvement can be achieved in the different domains for both groups. Indeed, after the treatment, no significant differences in the different measures were present between the two groups. Thus, these results replicate other findings indicating that even in chronic aphasia, language improvements can be achieved through intensive therapy [60, 61] which makes use of a pragmatic approach, such as conversational therapy [12–16]. Moreover, the fact that there was no difference between the VR group and the conventional therapy group suggests, in accordance with previous studies [see 44] good compatibility and feasibility of the VR version. Interestingly, the within-subject comparisons revealed that the amount of improvement found in the different areas was distributed over far more language, communicative, and psychological aspects in the VR group than in the control group. Indeed, the VR training had a positive impact on three out of six tasks of the AAT test (repetition, written language, and oral comprehension), while in the control group, only on the repetition task. With regard to the C.A.P.P.A. test, the conversational approach resulted efficacious on the PWA communicative abilities independent of the presence of a VR support but it impacted across the different areas (i.e., language ability, self-correction, and turn taking) only in the VR group. As reported in Introduction, in the past, several VR systems have been developed for cognitive rehabilitation; some of which have only gone through studies with a small number of participants [43, 47, 48] and/or without control groups [32]. Most of the existing studies with VR-based cognitive rehabilitation focused on specific language domains [25–28], such as word retrieval [18, 19, 43, 44, 46–48], sentence building [20, 21], and language comprehension [22]. So NeuroVR was developed to target rehabilitation of multiple cognitive domains (i.e., language, verbal communication, attention, concentration, memory, and executive functions) simultaneously requiring the execution of daily routines in progressive levels of cognitive complexity.

The impact of VR for language recovery is in line with recent proposals from the embodied theory which considers language as represented in a multimodal dimension in which word semantics are also made of sensorimotor properties [62–68]. Thus, in order to facilitate language, it is efficacious to recreate a multimodal experience, such as the one that can be implemented through VR technology. Interestingly, self-reported data in the VASES and WHOQoL test revealed that, after the training, the VR group improved significantly in different psychological aspects such as their self-esteem, health, emotional, and humoral states and in their ability to maintain attention and concentration. These findings are especially relevant because our VR intervention targeted cognitive aspects but also improved the patients' emotional condition which is rarely taken into account when planning a language intervention. Indeed, the emotional and humoral states have consistently been associated with psychological well-being [69–74] and various authors have stressed that deficiencies in these psychological aspects are a crucial component in the development and course of depression in aphasia [75–78]. Given the high prevalence of depression following stroke (ranging from 25 to 79%, [77–79]) and the significant changes in physical, cognitive, and psychosocial functioning potentially experienced by the survivor [73, 74], it is easy to understand that stroke has a negative impact on psychological well-being. Indeed, the few available studies which have addressed this issue have suggested that a key personal resource contributing to psychosocial functioning after acquired brain injury is self-esteem [80–82]. Because the vast majority of instruments for mood evaluation are linguistically demanding, they have been of limited use in PWA. Consequently, right now, we know considerably less about how PWA feel than about how other stroke survivors feel. Most research on mood post stroke has either excluded PWA or relied on caregivers or health care staff to speak for them [80, 82]. In the present study, together with the WHOQoL, we used VASES [56] which is a reliable measure for identifying patients with a high risk for emotional dysfunction and in research on self-esteem after stroke due to the nonverbal nature of the test. Indeed, VASES [56] has been proven useful in identifying stroke survivors most at risk for emotional dysfunction and may be useful as a research tool in this population [56]. In our VR group, after the training, a significant increase in the level of self-esteem was found. Most probably, the possibility for the PWA to practice their communication skills within real situations and, above all, to interact among each other, has increased the patients' self-esteem helping them to overcome their language difficulties.

## 5. Conclusion

Overall, the results of this six-month study have revealed that language rehabilitation through an ecologically valid VR system can have a large impact in cognitive and psychological functioning. Thus, our results contribute with new evidence and provide further understanding on the use of VR in the rehabilitation of cognitive deficits. Despite the positive impact, some limitations of our study must be considered when interpreting the results. Concerning the sample, it can be observed that eighteen participants are still a small sample, though it is larger than previous studies. Moreover, the time post stroke comparison revealed that, although all patients were in the chronic phase (>36 months), the VR group was less chronic than the control group. Thus, there is still a need of further research considering other clinical populations, larger sample sizes, and more comparative studies. However, given the importance of a positive psychological state in PWA for motivating their participation in the therapy sessions, we believe that the use of VR, in the near future, should be pursued.

## Figures and Tables

**Table 1:** Demographic and clinical data of the thirty-six participants.

Participants	Age	Sex	Educational level	Time post onset	Etiology
S1	71	M	18	30	Frontotemporal hemorrhage
S2	50	F	8	28	Frontoparietal ischemia
S3	72	M	8	30	Frontotemporal ischemia
S4	68	M	13	40	Frontotemporal ischemia
S5	69	F	8	41	Frontal ischemia
S6	49	F	18	48	Temporoparietal hemorrhage
S7	53	M	13	36	Frontotemporal ischemia
S8	53	M	13	34	Frontoparietal ischemia
S9	71	M	13	54	Temporal ischemia
S10	32	M	15	40	Basal ganglia hemorrhage
S11	37	M	11	35	Temporoparietal hemorrhage
S12	51	M	13	30	Frontotemporal ischemia
S13	61	M	8	24	Temporoparietal ischemia
S14	48	M	8	24	Frontal hemorrhage
S15	72	F	5	30	Temporooccipital hemorrhage
S16	48	M	8	40	Frontal hemorrhage
S17	75	M	13	60	Temporoparietal ischemia
S18	70	M	8	30	Frontoparietal ischemia
S19	60	M	18	40	Frontotemporoparietal ischemia
S20	69	M	13	36	Frontotemporal ischemia
S21	56	F	13	35	Frontotemporal ischemia
S22	60	F	8	28	Temporal ischemia
S23	61	F	13	40	Frontotemporal ischemia
S24	53	M	13	42	Frontotemporal ischemia
S25	47	F	18	50	Frontotemporal ischemia
S26	61	M	13	54	Frontotemporal ischemia
S27	63	F	8	52	Frontotemporal hemorrhage
S28	70	F	8	60	Frontotemporal ischemia
S29	61	M	13	70	Frontotemporal ischemia
S30	38	M	13	54	Temporooccipital ischemia
S31	69	M	8	60	Frontotemporal ischemia
S32	70	M	8	58	Temporoparietal hemorrhage
S33	63	M	8	56	Frontotemporal ischemia
S34	60	M	9	52	Frontal ischemia
S35	77	F	13	50	Temporoparietal ischemia
S36	63	F	7	48	Temporoparietal ischemia

**Table 2:** Virtual scenarios.

Station Patients explore the Porta Nuova railway station (Turin). They must proceed with the purchase of the railway ticket by providing their personal information, check the train track, and manage possible unexpected events (e.g., the train display board is not working, facing a stranger who is asking for help because he was robbed, and managing the seat occupied by another passenger).
Hotel Patients are in the hotel, they have to check-in, decide how many days to stay, ask for breakfast time, and how to set the alarm clock. They must also decide which room they want and find their way around the hotel. In addition, they must manage possible unexpected events (e.g., a broken glass, a forgotten suitcase, and a mouse in the room).
Restaurant Patients must initially make a phone reservation to reserve a table in the restaurant. At the restaurant, they have to choose what they want from the menu, order from the waiter, and pay the bill. In addition, they must handle possible unexpected events concerning the payment of the bill and the dishes ordered.
Supermarket Patients must shop inside a supermarket with reference to a list of foods. They must, therefore, ask information to the clerks, choose the products, check that they have purchased everything, and go to the cash register. A possible unexpected event is represented by a thief who steals the wallet of an elderly lady. Patients must help the lady by contacting the police.
Amusement park Patients are located inside an amusement park. They must go to the entrance desk, ask for tickets, decide on which rides to climb, and face possible unexpected events (e.g., they lose some objects while getting on the Ferris wheel).
Cinema Patients are watching a movie: “Cinderella”, and they are asked to tell the story and comment on the movie.
Travel Patients must take a trip that will take them on a cruise to Egypt. During the cruise, they will be able to perform various sports (e.g., tennis). In Egypt, they will visit several archaeological sites.

**Table 3:** Summary of the results obtained in the different subtests of the AAT test in the two groups.

Tests	VR group	Partial *η*^2^	Control group	Partial *η*^2^
AAT—token test	0.018	0.385	0.094	0.216
AAT—repetition	0.002^∗∗^	0.559	0.004^∗∗^	0.505
AAT—written language	0.002^∗∗^	0.552	0.045	0.296
AAT—naming	0.016	0.393	0.065	0.256
AAT—comprehension	0.027	0.347	0.019	0.382
AAT—oral comprehension	0.008^∗∗^	0.462	0.428	0.053
AAT—written comprehension	0.699	0.013	0.102	0.208

Sig. within-group ANOVA: ^∗∗^*p* ≤ 0.008.

**Table 4:** Summary of the results obtained in the different subtests of the C.A.P.P.A. test, for frequency and severity, in the VR and control groups from the patient's perspective.

	VR group	Partial *η*^2^	Control group	Partial *η*^2^
Frequency				
(i) Language ability	0.001^∗∗^	0.625	0.032	0.328
(ii) Self-correction	0.234	0.116	0.229	0.118
(iii) Turn taking	0.003^∗∗^	0.527	0.102	0.207
(iv) Topic management	0.278	0.097	0.001^∗∗^	0.685
Severity				
(i) Language ability	0.001^∗∗^	0.669	0.021	0.372
(ii) Self-correction	0.092	0.218	0.021	0.370
(iii) Turn taking	0.001^∗∗^	0.810	0.050	0.284
(iv) Topic management	0.046	0.291	0.034	0.322

Sig. within-group ANOVA: ^∗∗^*p* ≤ 0.008.

**Table 5:** Summary of the results obtained in the different subtests of the C.A.P.P.A. test, for frequency and severity, in the VR group and in the control group from the caregiver's perspective.

	VR group	Partial *η*^2^	Control group	Partial *η*^2^
Frequency				
(i) Language ability	0.001^∗∗^	0.740	0.011	0.432
(ii) Self-correction	0.001^∗∗^	0.634	0.001^∗∗^	0.606
(iii) Turn taking	0.001^∗∗^	0.662	0.085	0.227
(iv) Topic management	0.015	0.431	0.881	0.002
Severity				
(i) Language ability	0.001^∗∗^	0.677	0.001^∗∗^	0.614
(ii) Self-correction	0.003^∗∗^	0.578	0.020	0.373
(iii) Turn taking	0.011	0.456	0.189	0.139
(iv) Topic management	0.062	0.281	0.230	0.118

Sig. within-group ANOVA: ^∗∗^*p* ≤ 0.008.

**Table 6:** Summary of the results obtained in the VASES and WHOQoL test for the VR group and the control group.

	VR group	Partial *η*^2^	Without VR group	Partial *η*^2^
VASES	0.004^∗∗^	0.512	0.136	0.175
WHO physical	0.002^∗∗^	0.556	0.629	0.228
WHO social	0.097	0.212	0.003^∗∗^	0.525
WHO psychological	0.001^∗∗^	0.608	0.737	0.010
WHO environmental	0.001^∗∗^	0.720	0.114	0.194

Sig. within-group ANOVA ^∗∗^*p* ≤ 0.008.

## Data Availability

Data will be available upon request.
